# Cytotoxicity, Intestinal Transport, and Bioavailability of Dispersible Iron and Zinc Supplements

**DOI:** 10.3389/fmicb.2017.00749

**Published:** 2017-04-28

**Authors:** Hyeon-Jin Kim, Song-Hwa Bae, Hyoung-Jun Kim, Kyoung-Min Kim, Jae Ho Song, Mi-Ran Go, Jin Yu, Jae-Min Oh, Soo-Jin Choi

**Affiliations:** ^1^Division of Applied Food System, Major of Food Science and Technology, Seoul Women’s UniversitySeoul, South Korea; ^2^Department of Chemistry and Medical Chemistry, College of Science and Technology, Yonsei UniversityWonju, South Korea

**Keywords:** surface modification, SunActive^TM^, toxicity, uptake, oral absorption, dispersibility

## Abstract

Iron or zinc deficiency is one of the most important nutritional disorders which causes health problem. However, food fortification with minerals often induces unacceptable organoleptic changes during preparation process and storage, has low bioavailability and solubility, and is expensive. Nanotechnology surface modification to obtain novel characteristics can be a useful tool to overcome these problems. In this study, the efficacy and potential toxicity of dispersible Fe or Zn supplement coated in dextrin and glycerides (SunActive Fe^TM^ and SunActive Zn^TM^) were evaluated in terms of cytotoxicity, intestinal transport, and bioavailability, as compared with each counterpart without coating, ferric pyrophosphate (FePP) and zinc oxide (ZnO) nanoparticles (NPs), respectively. The results demonstrate that the cytotoxicity of FePP was not significantly affected by surface modification (SunActive Fe^TM^), while SunActive Zn^TM^ was more cytotoxic than ZnO-NPs. Cellular uptake and intestinal transport efficiency of SunActive Fe^TM^ were significantly higher than those of its counterpart material, which was in good agreement with enhanced oral absorption efficacy after a single-dose oral administration to rats. These results seem to be related to dissolution, particle dispersibility, and coating stability of materials depending on suspending media. Both SunActive^TM^ products and their counterpart materials were determined to be primarily transported by microfold (M) cells through the intestinal epithelium. It was, therefore, concluded that surface modification of food fortification will be a useful strategy to enhance oral absorption efficiency at safe levels.

## Introduction

Iron deficiency is the most common nutritional disorder often occurs in infants, children, and premenopausal women, causing iron deficiency anemia. Approximately, 20–50% of the world population are reported to be affected by iron deficiency (Beard and Stoltzfus, 2001; Hider and Kong, 2013). Zinc deficiency is another important nutrition-related health problem, which affects about 25% of population around the world (Prasad, 2012; Raya et al., 2016). Since a small portion of iron or zinc in the diet can be absorbed, the deficiencies are mainly associated with low bioavailability of these elements.

Food fortification refers to the addition of essential trace elements and vitamins to food, so as to improve the nutritional quality of the food and to provide a public health benefit with minimal risk to health [World Health Organization (WHO) and Food and Agriculture Organization (FAO)]. This is a relatively simple and efficient way to prevent mineral deficiency. However, mineral fortification in food has challenges because it often causes unacceptable organoleptic changes during preparation process and storage, has a low bioavailability, and is expensive (Hurrell, 2002). For example, water-soluble ferrous sulfate has a good bioavailability, but induces unacceptable color or flavor changes in food (Hurrell, 1997). In contrast, ferric pyrophosphate (FePP) does not induce serious organoleptic change, although it has low bioavailability coming from poor water solubility (Kloots et al., 2004). In case of Zn fortification, zinc sulfate, zinc oxide (ZnO), zinc acetate, and zinc gluconate have been frequently used (Brown and Wuehler, 2000; Brown et al., 2004). Recently, zinc nanoparticle (NP) formulation, like ZnO-NP, has also attracted much attention to enhance intestinal absorption and bioavailability and to reduce undesirable side effects, taking advantage of high surface area to volume ratio and great reactivity as nano-sized materials (Srinivas et al., 2010; Torabi et al., 2013; Zhao et al., 2014; Faiz et al., 2015; Raya et al., 2016). However, ZnO-NP itself tends to be easily precipitated or aggregated, thereby possessing low stability under physiological condition. Moreover, most forms for Zn fortification have unpleasant zinc flavor when applied in the diet.

A modification of surface characteristics can be a fascinating strategy to overcome such disadvantages in food fortification, for example, by using colloidal techniques, emulsification, surface coating, and etc. (Rossi et al., 2014). In this regard, innovative SunActive^TM^ compounds (Taiyo Kagaku, Yokkaichi, Japan), in which FePP or ZnO-NPs are emulsified by dextrin, surfactants, and lecithin (Nanbu et al., 2000; Honda et al., 2010), are currently available on the market. These techniques provide high dispersion stability in aqueous media, and thus, can be also used for application in liquid foods or drinks without compromising taste. It was reported that SunActive Fe^TM^, an emulsified FePP with dextrin and glycerides, has a similar bioavailability to ferrous sulfate when added to a wheat-milk infant cereal and a yogurt drink (Fidler et al., 2004). Similarly, SunActive Zn^TM^, an emulsion of ZnO-NPs with maltodextrin and glycerides, was reported to enhance Zn bioavailability in zinc-deficient rats (Ishihara et al., 2008).

Nanotechnology application to food sectors covers techniques or tools used during cultivation, production, processing, or packaging of the food, as defined by the Nanoforum (Joseph and Morrison, 2006). Surface modification of Fe or Zn moiety by SunActive^TM^ coating, which induces novel characteristics, can be an important nanotechnology strategy, although an average particle size of SunActive^TM^ products have been reported to be relatively large (∼300 nm) (Fidler et al., 2004; Sakaguchi et al., 2004; Ishihara et al., 2008; Roe et al., 2009). Nevertheless, SunActive^TM^ coating can be classified as a nano-coating, considering general definition of nanomaterials as “particles with dimensions less than one micrometer (i.e., <1000 nm) that exhibit unique properties not recognized in micron or larger sized particles,” provided by US Food and Drug Administration (Food and Drug Administration [FDA], 2014).

Relatively little information is currently available on physicochemical characteristics and *in vitro* and *in vivo* efficacy as well as toxicity of commercially available, nanotechnology applied mineral fortification products. The aim of the present study was, therefore, to answer the question as to whether they have enhanced efficacy, without affecting potential toxicity. We evaluated the cytotoxicity of SunActive Fe^TM^ and SunActive Zn^TM^ in human intestinal cells. Furthermore, the efficacy and bioavailability of SunActive^TM^ products were assessed in terms of cellular uptake, intestinal transport mechanism, and *in vivo* biokinetics. The physicochemical properties of SunActive^TM^ products, such as solubility, dispersibility, and coating stability, were also analyzed to explain their biological behaviors. In all experiments, FePP and ZnO-NPs with similar primary particle size compared to SunActive^TM^ products were used as counterpart materials for comparative study.

## Materials and Methods

### Materials

SunActive Fe^TM^ (FePP 26.6%, dextrin 54.7%, glycerides 5.3%, lecithin 1.3%, and etc.) and SunActive Zn^TM^ (ZnO 30.0%, maltodextrin 62.1%, glyceride 6.0%, lecithin 1.9%, and etc.) were purchased from Taiyo Kagaku (Yokkaichi, Japan). According to the certificate of analysis provided by the manufacturer, both Sunactive Fe^TM^ and Sunactive Zn^TM^ were prepared by materials mixing, pasteurization at 80°C, homogenization, filtration, and spray drying. FePP and ZnO-NPs possessing similar primary particle sizes to corresponding SunActive^TM^ product were purchased from Dr. Paul Lohmann GmbH KG (Emmerthal, Germany) and American Elements, Co. Ltd (Los Angeles, CA, USA), respectively.

### Characterization

Crystalline phase of each sample was analyzed by powder X-ray diffractometry (XRD, Bruker AXS D2 Phaser, Billerica, MA, USA) with Cu Kα radiation (λ = 1.5418 Å). The powder sample mounted on poly (methyl methacrylate) holder was scanned from 5 to 80° (2θ) with 0.1 mm equatorial slit and 1.0 mm air scattering slit. For measurement of hydrodynamic radius of materials, aqueous suspension of each sample (1 mg/ml) was subjected to dynamic light scattering (DLS) instrument (ELSZ-1000, Otsuka, Japan). The particle size and morphology of powder samples were examined with field emission-scanning electron microscope (SEM; Quanta 250 FEG, FEI Company, Hillsboro, OR, USA). Each powder was loaded on carbon tape and coated with Pt/Pd for 60 s before SEM measurement.

### Solubility and Hydrodynamic Radii in Cell Culture Medium and Simulated Body Fluids

Dissolution of metal species from each sample was evaluated in cell culture minimum essential medium (MEM; Welgene, Gyeongsangbuk-do, South Korea) and simulated body fluids, such as gastric and intestinal solutions. For preparation of simulated gastric solution, 4 g NaCl (Daejung Chemicals & Metals, Gyeonggi-do, South Korea) and 6.4 g pepsin (Sigma–Aldrich, St. Louis, MO, USA) were dissolved in 1,900 ml of distilled and deionized water (DDW), and then the solution was titrated with 1 M HCl until pH 1.5. In order to prepare simulated intestinal solution, pH of simulated gastric solution was adjusted to 6.8 with 0.95 M NaHCO_3_ (Sigma–Aldrich) solution, and then, 175 mg of bile salt (Sigma–Aldrich) and 50 mg pancreatin (Sigma–Aldrich) were added.

Each sample with designated mass was dispersed in MEM containing 10% fetal bovine serum (FBS), and simulated gastric or intestinal solution. The final concentrations of suspension were 3.72, 1, 3.24, and 1 mg/ml for SunActive Fe^TM^, FePP, SunActive Zn^TM^, and ZnO-NPs, respectively, to have an equivalent Zn or Fe amount. Each SunActive^TM^ suspension was prepared to contain an equivalent Fe or Zn amount compared to its counterpart, FePP or ZnO-NPs. After incubation for 24 h, the supernatants were collected by centrifugation (9,240 × *g*), pre-treated with ultrapure nitric acid, filtered through syringe filter (0.45 μm, Advantec MFS, Inc., Dublin, CA, USA), and then subjected to inductively coupled plasma-atomic emission spectroscopy (JY2000 Ultrace, HORIBA Jobin Yvon, Longjumeau, France).

In order to investigate dispersibility of samples under physiological conditions, hydrodynamic radii in MEM and simulated body fluids were measured. Each suspension was prepared to have 1 mg/ml concentration, and then subjected to DLS measurement (ELSZ-1000).

### Cell Culture

Human intestinal epithelial INT-407 cells were provided by Dr. Tae-Sung Kim at Korea University (Seoul, South Korea) and cultured in MEM (Welgene). Caco-2 cells were purchased from the Korean Cell Line Bank (Seoul, South Korea) and cultured in Dulbecco’s Modified Eagle Medium (DMEM; Welgene), under a humidified atmosphere (5% CO_2_/95% air) at 37°C. The media were supplemented with 10% heat inactivated FBS, 100 units/ml of penicillin, and 100 μg/ml of streptomycin (Welgene).

### Cell Proliferation

The effect of materials on cell proliferation was measured with the water soluble tetrazolium salt-1 (WST-1; Roche, Molecular Biochemicals, Mannheim, Germany). Cells (5 × 10^3^ cells/100 μl) were incubated with SunActive^TM^ products or equivalent amounts of FePP or ZnO-NPs based on Fe or Zn content for 24 h. Then, 10 μl of WST-1 solution (Roche) was added to each well, and cells were further incubated for 4 h. Absorbance was measured using a plate reader at 440 nm (SpectraMax^®^ M3, Molecular Devices, Sunnyvale, CA, USA). Cells incubated in the absence of materials were used as controls.

### Clonogenic Assay

Cells (5 × 10^2^ cells/2 ml) were seeded in 6-well plates and incubated overnight at 37°C under a 5% CO_2_ atmosphere. The medium in the plates was then replaced with fresh medium containing various concentrations of SunActive^TM^ materials or equivalent amounts of FePP or ZnO-NPs based on Fe or Zn content, and incubation was continued for 7 days. For colony counting, cells were washed with phosphate buffered saline (PBS) and fixed with 90% crystal violet solution (Sigma–Aldrich) for 1 h. After cell washing with DDW and air-drying, colonies consisted of more than 50 cells were counted. Each experiment was done in triplicate and colony numbers in the absence of test materials were used as controls.

### LDH Leakage Assay

The release of lactate dehydrogenase (LDH) was monitored with the CytoTox 96 Non-Radioactive Cytotoxicity assay (Promega, Madison, WI, USA). Cells (2 × 10^4^ cells/1 ml) were exposed to SunActive^TM^ products or equivalent amounts of FePP or ZnO-NPs based on Fe or Zn content for 24 h. The cells were detached with trypsin- ethylenediaminetetraacetic acid (EDTA) treatment, centrifuged, and aliquots (50 μl) of cell culture medium were collected from each well and placed in new microplates. Then, 50 μl of substrate solution was added to each well and the plates were further incubated for 30 min at room temperature. Finally, after adding 50 μl of stop solution, the absorbance at 490 nm was measured with a microplate reader (SpectraMax^®^ M3, Molecular Devices). Cytotoxicity is expressed relative to the basal LDH release from untreated control cells.

### Intracellular ROS Production

Intracellular reactive oxygen species (ROS) levels were monitored using a peroxide-sensitive fluorescent probe, 2′,7′-dichlorofluorescein diacetate (H_2_DCFDA) (Molecular Probes, Inc., Eugene, OR, USA), according to the manufacturer’s guidelines. Briefly, cells (1 × 10^4^ cells/100 μl) were incubated with SunActive^TM^ products or equivalent amounts of FePP or ZnO-NPs based on Fe or Zn content for 24 h under standard condition as described above, washed with PBS, and incubated with 5 μM H_2_DCFDA for 60 min at 37°C. After washing with PBS, DCF fluorescence was immediately measured using a fluorescence microplate reader (SpectraMax^®^ M3, Molecular Devices). Excitation and emission wavelengths were 495 and 518 nm, respectively. Cells not treated with test materials were used as controls.

### Cellular Uptake

Cells (1 × 10^6^ cells/2 ml) were incubated overnight at 37°C, and then, the medium was replaced with fresh medium containing SunActive^TM^ products or an equivalent amount of FePP or ZnO-NPs (50 μg/ml Fe or Zn content). After 6 h of incubation, the cells were washed three times with PBS, treated with 5 mM EDTA in PBS for 40 s to detach adsorbed particles on the cell membrane, and further washed with PBS. The cells were harvested by scraping and re-suspended in DDW. After centrifugation, the cell pellets were digested and Fe or Zn concentrations were determined by ICP-AES (JY2000 Ultrace, HORIBA Jobin Yvon), as described in “ICP-AES analysis” below.

### Intestinal Transport Mechanism

#### 3D Cell Culture for Intestinal Epithelial Monolayers

The monoculture system of Caco-2 cells, representing the intestinal epithelium monolayers of tight junctions, was prepared as follow; after coating Transwell^®^ inserts (SPL Life Science, Gyeonggi-do, South Korea) with Matrigel^TM^ matrix (Becton Dickinson, Bedford, MA, USA) for 1 h, supernatants were removed, and then inserts were washed with DMEM. Caco-2 cells (4.5 × 10^5^ cells/well) were grown on upper insert sides, cultured for 21 days, and treated with SunActive^TM^ products or an equivalent amount of FePP or ZnO-NPs (50 μg/ml Fe or Zn content) for 6 h. The concentrations of transported Fe or Zn in basolateral solutions were determined by ICP-AES (JY2000 Ultrace, HORIBA Jobin Yvon).

#### 3D Cell Culture for FAE Model

Non-adherent human Burkitt’s lymphoma Raji B cells were purchased from the Korean Cell Line Bank and grown in RPMI 1640 medium, supplemented with 10% FBS, 1% non-essential amino acids, 1% L-glutamine, 100 units/ml of penicillin, and 100 μg/ml of streptomycin at 37°C in 5% CO_2_ atmosphere. An *in vitro* model of human intestinal follicle-associated epithelium (FAE) was prepared according to the method described by des Rieux et al. (2007) to study material transport by microfold (M) cells; Caco-2 cells (1 × 10^6^ cells/well) were grown on upper insert sides in the same manner as described in Caco-2 monoculture system and incubated for 14 days. Raji B cells (1 × 10^6^ cells/well) in DMEM were then added to basolateral insert compartments, and these co-cultures were maintained for 5 days. Apical medium of cell monolayers was then replaced with a particle suspension containing SunActive^TM^ products or an equivalent amount of FePP or ZnO-NPs (50 μg/ml Fe or Zn content), and treated for 6 h. The concentrations of transported Fe or Zn in basolateral solutions were determined by ICP-AES (JY2000 Ultrace, HORIBA Jobin Yvon).

### Animals

Five-week-old female Sprague Dawley (SD) rats weighing 130–150 g were purchased from Nara Biotech, Co., Ltd (Seoul, South Korea). Animals were housed in plastic laboratory animal cages in a ventilated room maintained at 20 ± 2°C and 60 ± 10% relative humidity under a 12 h light/dark cycle. Water and commercial laboratory complete food were provided *ad libitum*. Animals were allowed to acclimate to the environment for 7 days before treatment. All animal experiments were performed in compliance with the guideline issued by the Animal and Ethics Review Committee of Seoul Women’s University and Ethics Review Committee of Seoul Women’s University approved the procedures used in this study (IACUC-2014A-3).

### Biokinetics and Tissue Distribution

Six female rats per group were administered a single dose of SunActive Fe^TM^ (1000 mg/kg) or SunActive Zn^TM^ (500 mg/kg) and an equivalent Fe or Zn amount of FePP (320 mg/kg) or ZnO-NPs (156.25 mg/kg) by oral gavage; controls (*n* = 6) received an equivalent volume of distilled water (DW). To determine plasma Fe or Zn concentrations, blood samples were collected *via* tail vein at 0, 0.25, 0.5, 1, 2, 4, 6, 10, and 24 h. Blood samples were centrifuged at 3,000 × *g* for 15 min at 4°C to obtain plasma. Plasma Fe or Zn concentration-time profiles were determined by ICP-AES (JY2000 Ultrace, HORIBA Jobin Yvon). The following biokinetic parameters were calculated using Kinetica version 4.4 (Thermo Fisher Scientific, Waltham, MA, USA): maximum concentration (C_max_), time to maximum concentration (T_max_), area under the plasma concentration-time curve (AUC), half-life (T_1/2_), and mean residence time (MRT).

For the tissue distribution study, the same doses were orally administered. Tissue samples of kidneys, liver, lungs, and spleen were collected after CO_2_ euthanasia at 24 h post-administration. Fe or Zn biodistribution was determined by ICP-AES (JY2000 Ultrace, HORIBA Jobin Yvon).

### ICP-AES Analysis

Biological samples were pre-digested with 10 ml of ultrapure nitric acid (HNO_3_) overnight, heated to ∼160°C, and 1 ml of hydrogen peroxide (H_2_O_2_) solution was added. The mixtures were heated until the samples were colorless and clear. After dilution with 3 ml of DDW, total Fe or Zn concentrations were determined by ICP-AES (JY2000 Ultrace, HORIBA Jobin Yvon).

### Statistical Analysis

Results were expressed as means ± standard deviations. Experimental values were compared with corresponding untreated control values. One-way analysis of variance (ANOVA) with Tukey’s test in SAS Version 9.4 (SAS Institute, Inc., Cary, NC, USA) was used to determine the significances of intergroup differences. Statistical significance was accepted for *p* values of less than 0.05.

## Results

### Physicochemical Properties

**FIGURE 1:**
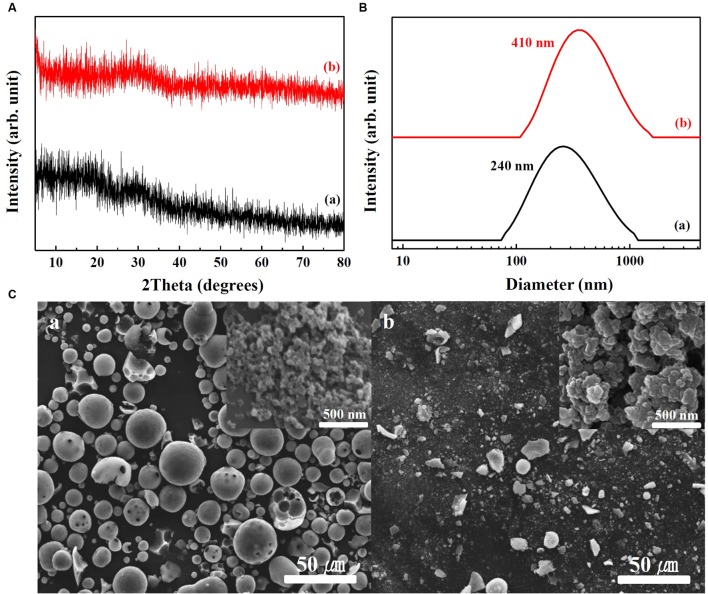
**(A)** XRD patterns, **(B)** hydrodynamic radii measured by DLS, and **(C)** SEM images of (a) SunActive Fe^TM^ and (b) FePP. Insets in (**C**-a,b) are magnified images at the surface of materials.

**FIGURE 2:**
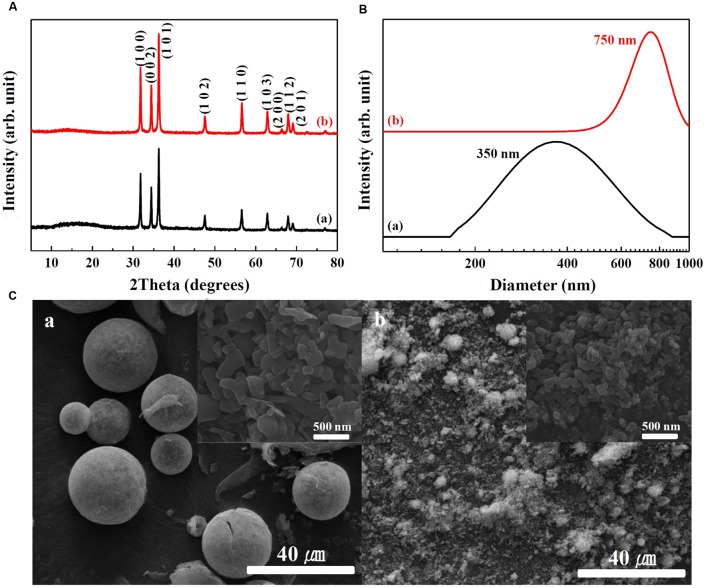
**(A)** XRD patterns, **(B)** hydrodynamic radii measured by DLS, and **(C)** SEM images of (a) SunActive Zn^TM^ and (b) ZnO-NPs. Insets in (**C**-a,b) are magnified images at the surface of materials.

Physicochemical properties of SunActive Fe^TM^ and SunActive Zn^TM^ are displayed in **Figures 1**, **2**, respectively. XRD results show that both SunActive Fe^TM^ and FePP had amorphous phase (**Figure 1A**). Hydrodynamic radii, representing the secondary particle sizes in DDW suspension, were 240 and 410 nm for SunActive Fe^TM^ and FePP (**Figure 1B**), respectively. The SEM images in low magnification exhibited sphere-like morphology for SunActive Fe^TM^, which seems to be attributed to the organic moiety, such as dextrin and glycerides, while FePP showed irregular aggregation (**Figure 1C**). Magnified images taken at the surface of materials showed comparable primary particle size of ∼50 nm for both SunActive Fe^TM^ and FePP (insets in **Figure 1C**). On the other hand, both SunActive Zn^TM^ and ZnO-NPs had the typical Wurtzite crystal phase [**Figure 2A**, Joint Committee on Powder Diffraction Standards (JCPDS) No. 36-1451], indicating the existence of ZnO-NPs in SunActive Zn^TM^. Hydrodynamic radii of SunActive Zn^TM^ and ZnO-NPs were 350 and 750 nm, respectively, indicating higher dispersion degree of SunActive Zn^TM^ (**Figure 2B**). Similar to SunActive Fe^TM^, SunActive Zn^TM^ showed spherical morphology in low magnification, while ZnO-NPs had irregular and agglomerated shape (**Figure 2C**). Magnified SEM images at the surface of materials exhibited fairly similar primary particle size of 112 ± 42 and 103 ± 20 nm for SunActive Zn^TM^ and ZnO-NPs, respectively (insets in **Figure 2C**).

### Solubility and Hydrodynamic Radii in Cell Culture Medium and Simulated Body Fluids

Dissolution properties of Fe or Zn from SunActive Fe^TM^ or SunActive Zn^TM^ in MEM and simulated gastric or intestinal solutions were compared to those of their counterpart materials (FePP or ZnO-NPs) and summarized in **Table 1**. There were no significant differences in Fe dissolution between SunActive Fe^TM^ and FePP in MEM, although slightly high solubility was found for FePP in simulated gastric fluid. On the other hand, Fe solubility from SunActive Fe^TM^ significantly and remarkably increased compared to FePP under intestinal condition. Similar to Fe samples, Zn dissolution from SunActive Zn^TM^ was similar to that from ZnO-NPs in both MEM and gastric solution. Whereas, Zn solubility was slightly higher for ZnO-NPs than SunActive Zn^TM^ in intestinal solution, and both Zn samples showed the highest solubility in gastric solution. In other words, solubilities between SunActive^TM^ products and their counterparts were similar in MEM, while statistically different solubility was found in simulated intestinal solution.

**Table 1:** Dissolution properties of Fe or Zn from SunActive^TM^ products and their counterpart materials in cell culture medium (MEM) and simulated body fluids.

Materials	Unit	MEM		Simulated gastric solution		Simulated intestinal solution	
				
		Fe	Zn	Fe	Zn	Fe	Zn
SunActive Fe^TM^	% (w/w)	0.067 ± 0.75		2.47 ± 0.20^a^		25.6 ± 2.68^a^	
FePP	% (w/w)	N.D.		4.02 ± 0.64^b^		0.27 ± 0.14^b^	
SunActive Zn^TM^	% (w/w)		2.24 ± 0.42		86.0 ± 0.58		1.48 ± 0.11^a^
ZnO-NP	% (w/w)		1.87 ± 0.77		84.1 ± 1.19		1.90 ± 0.03^b^


*Mean values with different superscripts (a,b) in the same column indicate significant difference between SunActive^*TM*^ product and its counterpart (*p* < 0.05).*

*N.D., not determined; MEM, minimum essential medium; FePP, ferric pyrophosphate.*

**FIGURE 3:**
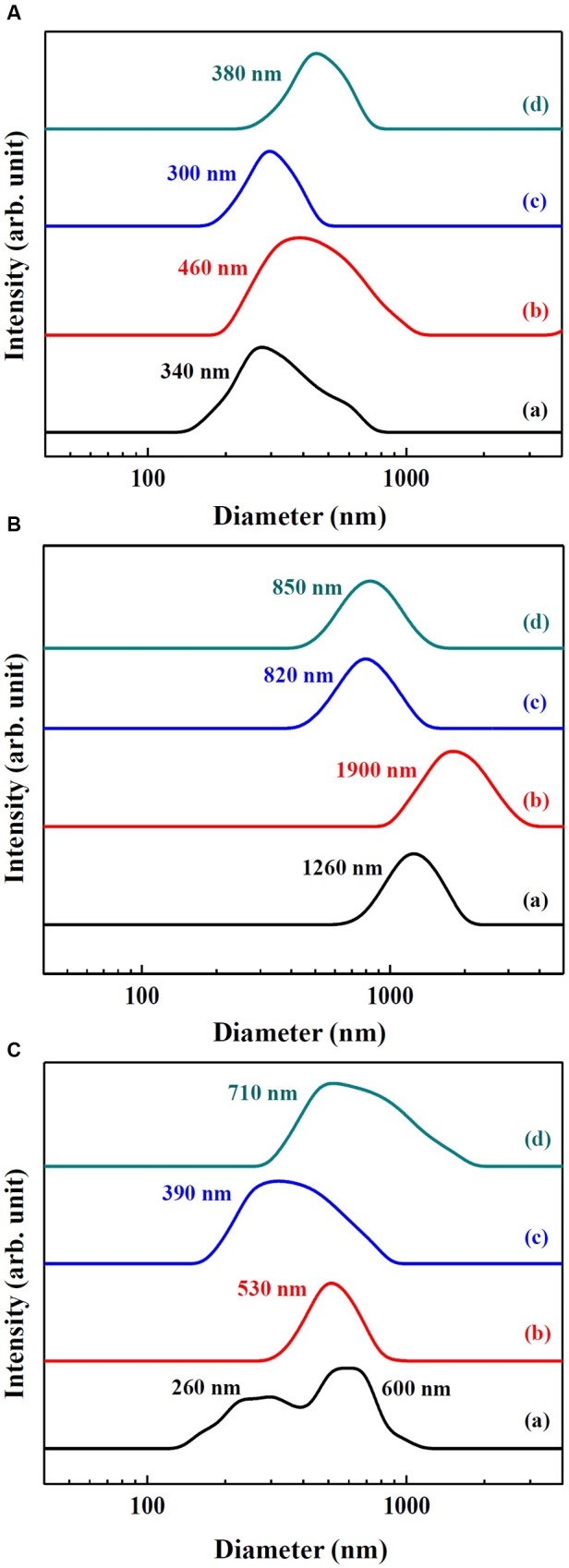
**Hydrodynamic radius of (a) SunActive Fe^TM^, (b) FePP, (c) SunActive Zn^TM^, and (d) ZnO-NPs in** **(A)** MEM, **(B)** simulated gastric solution, and **(C)** simulated intestinal solution.

Hydrodynamic radii of SunActive^TM^ products and their counterpart materials in MEM and simulated gastric and intestinal solutions were measured by DLS and displayed in **Figure 3**. The hydrodynamic radii were found to be highly dependent on the type of medium. The hydrodynamic radii of SunActive Fe^TM^, FePP, and SunActive Zn^TM^ in MEM were 340, 460, and 300 nm, respectively (**Figure 3A**), which were comparable to the values obtained in DDW (**Figures 1B**, **2B**). The hydrodynamic radius of ZnO-NPs in MEM considerably reduced compared to that in DDW, showing 380 nm. Under simulated gastric condition, the hydrodynamic radii of all materials significantly increased and more prominent increase in size was found for Fe samples than Zn samples (**Figure 3B**). In simulated intestinal fluid (**Figure 3C**), both SunActive Fe^TM^ and FePP had increased hydrodynamic radii and reduced homogeneity compared to those in MEM (**Figure 3A**). Furthermore, SunActive Fe^TM^ showed split distribution in the range from 150 to 1000 nm. In case of SunActive Zn^TM^ and ZnO-NPs, the single distribution patterns and similar hydrodynamic radii were found in intestinal fluid, compared to those in DDW.

### Short- and Long-Term Cytotoxicity

**FIGURE 4:**
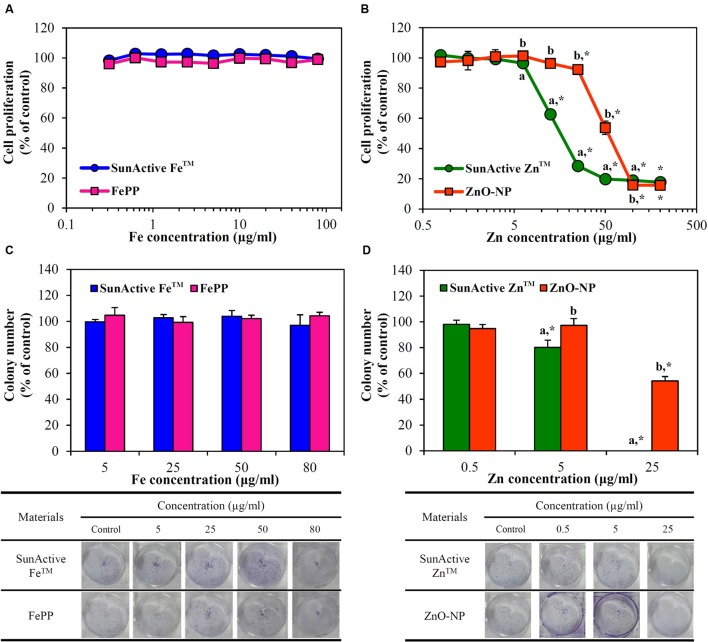
**Short-term effect of** **(A)** SunActive Fe^TM^ and **(B)** SunActive Zn^TM^ on cell proliferation of INT-407 cells after treatment for 24 h, measured by WST-1 assay. Long-term effect of **(C)** SunActive Fe^TM^ and **(D)** SunActive Zn^TM^ on colony-forming ability of INT-407 cells incubated for 7 days. FePP and ZnO-NPs were used as counterpart materials for SunActive Fe^TM^ and SunActive Zn^TM^, respectively. Mean values with different letters (a,b) in the same concentrations indicate significant differences between SunActive^TM^ product and its counterpart material (*p* < 0.05). ^∗^ Indicates significant differences from the untreated controls (*p* < 0.05).

Cytotoxicity of SunActive Fe^TM^ or SunActive Zn^TM^ was evaluated in human intestinal INT-407 cells in terms of inhibition of cell proliferation and colony-forming ability, and compared to that of each counterpart material. **Figure 4A** demonstrates that both SunActive Fe^TM^ and FePP did not affect cell proliferation and viability up to the highest concentration tested after exposure for 24 h. On the other hand, SunActive Zn^TM^ more remarkably inhibited cell proliferation than ZnO-NPs (**Figure 4B**). It is worth noting that all cytotoxicity tests were performed based on equivalent Fe or Zn amounts, and the counterpart materials had similar primary particle size to corresponding SunActive^TM^ products. It was determined that Fe and Zn contents in SunActive Fe^TM^ and SunActive Zn^TM^ are 8 and 25%, respectively, and therefore, the highest concentrations as total compounds for the former and the latter were 1000 μg/ml (equivalent to 80 μg/ml Fe content) and 800 μg/ml (equivalent to 200 μg/ml Zn content), respectively.

Long-term cytotoxicity was also evaluated by clonogenic assay after treatment for 7 days, which investigates the ability of colony formation of cells. Interestingly, SunActive Fe^TM^ and FePP did not affect colony-forming ability at all (**Figure 4C**). Whereas, SunActive Zn^TM^ remarkably inhibited colony formation at the highest concentration tested (25 μg/ml), as compared to ZnO-NPs (**Figure 4D**). The same tendency was observed in human intestinal Caco-2 cells exposed to SunActive^TM^ products (data not shown).

### Membrane Damage and ROS Generation

**FIGURE 5:**
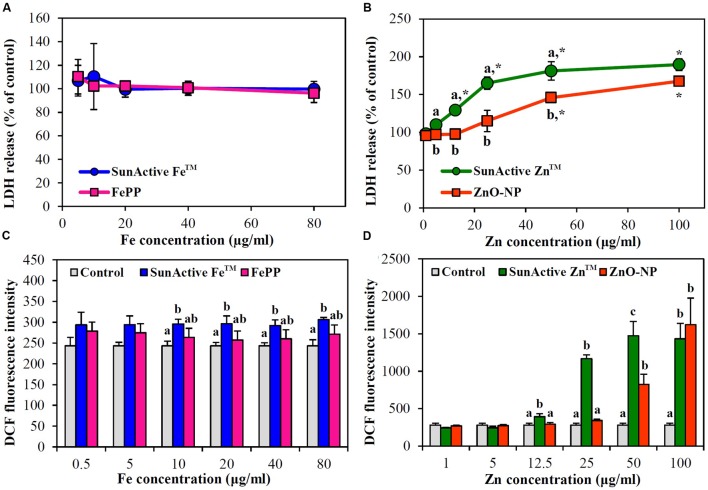
**Effect of** **(A,C)** SunActive Fe^TM^ and **(B,D)** SunActive Zn^TM^ on **(A,B)** LDH release and **(C,D)** ROS generation in INT-407 cells after treatment for 24 h. FePP and ZnO-NPs were used as counterpart materials for SunActive Fe^TM^ and SunActive Zn^TM^, respectively. Mean values with different letter (a,b,c) in the same concentrations indicate significant differences among untreated control, SunActive^TM^ product, and its counterpart material (*p* < 0.05). ^∗^ Indicates significant differences from the untreated controls (*p* < 0.05).

Membrane damage caused by SunActive^TM^ products was evaluated using LDH leakage assay, which measures released amount of cytosolic LDH into extracellular medium. The same tendency obtained from WST-1 assay was found; neither SunActive Fe^TM^ nor FePP did induce membrane damage (**Figure 5A**), while significantly high LDH leakage from SunActive Zn^TM^-exposed cells compared to ZnO-NP exposure was found (**Figure 5B**).

When intracellular ROS generation was measured with H_2_DCFDA, slightly but significantly increased ROS levels were detected by SunActive Fe^TM^ treatment, whereas, no elevated ROS levels were found in FePP-exposed cells (**Figure 5C**). Meanwhile, both SunActive Zn^TM^ and ZnO-NPs significantly induced ROS at Zn concentrations of more than 50 μg/ml, but much high ROS levels were induced by SunActive Zn^TM^ at concentration range from 12.5 to 50 μg/ml (**Figure 5D**). The same tendency was found in Caco-2 cells (data not shown).

### Cellular Uptake

**FIGURE 6:**
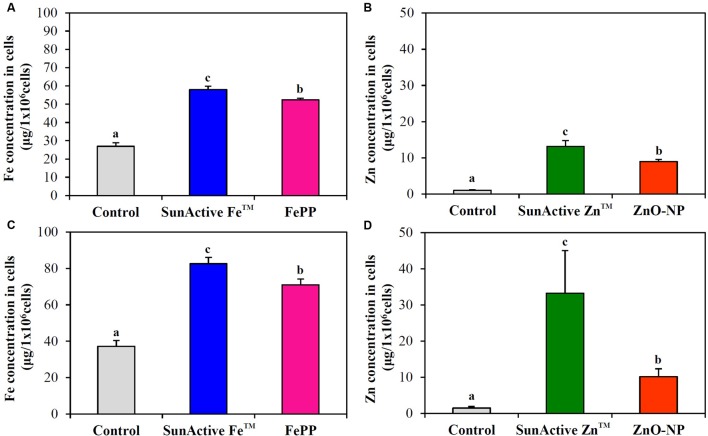
**Cellular uptake of** **(A,C)** SunActive Fe^TM^ and **(B,D)** SunActive Zn^TM^ in **(A,B)** Caco-2 cells and in **(C,D)** INT-407 cells after treatment for 6 h, measured by ICP-AES. FePP and ZnO-NPs were used as counterpart materials for SunActive Fe^TM^ and SunActive Zn^TM^, respectively. Mean values with different letters (a,b,c) in the same figure indicate significant differences among untreated control, SunActive^TM^ product, and its counterpart material (*p* < 0.05).

Cellular uptake efficacy of SunActive^TM^ products was evaluated in both INT-407 cells and Caco-2 cells by measuring intracellular Fe or Zn amount by ICP-AES, and compared with that of each counterpart material. **Figure 6** shows that both SunActive Fe^TM^ and SunActive Zn^TM^ were more efficiently taken up by cells than FePP and ZnO-NPs, respectively. Significantly, high uptake of both materials was found in INT-407 cells (**Figures 6C,D**) than in Caco-2 cells (**Figures 6A,B**).

### Intestinal Transport Mechanism

**FIGURE 7:**
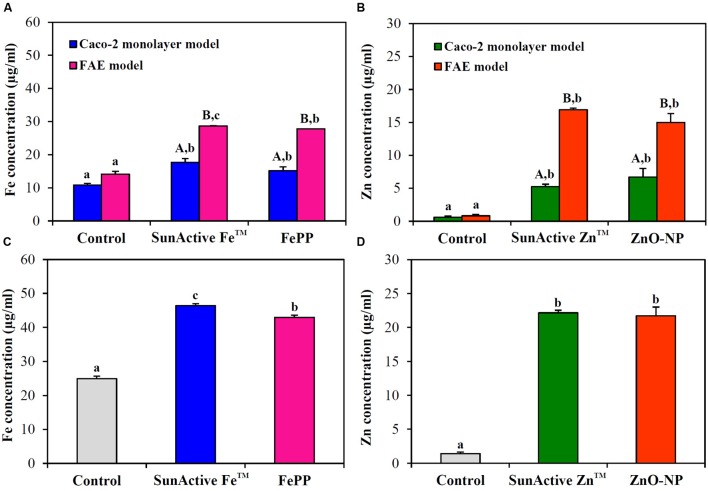
**Intestinal transport mechanism of** **(A)** SunActive Fe^TM^ and **(B)** SunActive Zn^TM^ evaluated using *in vitro* models of Caco-2 monolayers and human FAE after treatment for 6 h. Combined total intestinal transport amount of **(C)** SunActive Fe^TM^ and **(D)** SunActive Zn^TM^ through both Caco-2 monolayers and the FAE model. FePP and ZnO-NPs were used as counterpart materials for SunActive Fe^TM^ and SunActive Zn^TM^, respectively. Mean values with different letters in minuscule (a,b,c) in the same figure indicate significant differences among untreated control, SunActive^TM^ product, and its counterpart material (*p* < 0.05). Mean values with different letters in majuscule (A,B) in the same figure indicate significant differences between Caco-2 monolayers and the FAE model (*p* < 0.05).

Intracellular transport pathway was assessed using *in vitro* models of human FAE and Caco-2 monolayers, which represent M cells in Peyer’s Patches and intestinal tight junctions, respectively. The results demonstrate that both SunActive^TM^ materials were primarily transported by M cells, but their significantly elevated transports through Caco-2 monolayers were also found (**Figures 7A,B**). FePP and ZnO-NPs were also found to be translocated across both M cells and Caco-2 tight junctions. Total intestinal transport amounts of SunActive^TM^ materials through both M cells and Caco-2 monolayers were pooled and presented in **Figures 7C,D**. Significantly higher translocation of SunActive Fe^TM^ than FePP was found, contrary to SunActive Zn^TM^ showing similar total transport amount compared to ZnO-NPs.

### Biokinetics and Tissue Distribution

**FIGURE 8:**
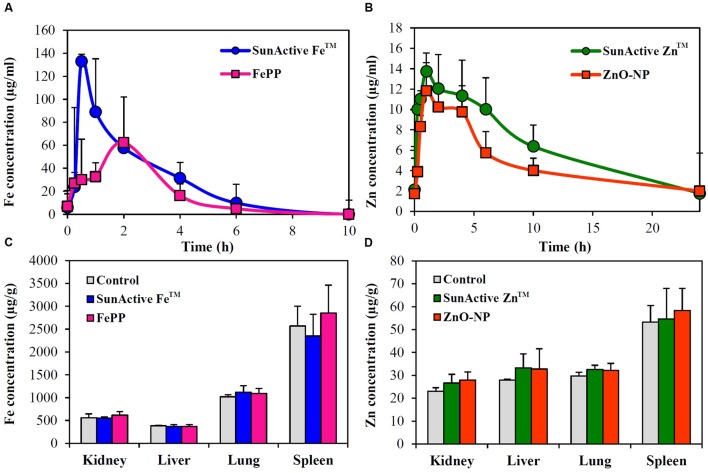
**Plasma concentration-time profiles of** **(A)** SunActive Fe^TM^ (1000 mg/kg) and **(B)** SunActive Zn^TM^ (500 mg/kg) after a single-dose oral administration to rats. An equivalent amount of FePP (320 mg/kg) or ZnO-NPs (156.25 mg/kg) based on Fe or Zn content was also orally administered for comparative study. Tissue distribution patterns of **(C)** SunActive Fe^TM^ (1000 mg/kg) and **(D)** SunActive Zn^TM^ (500 mg/kg) after a single-dose oral administration to rats at 24 h post-administration.

Plasma concentration versus time curves after a single-dose oral administration to rats demonstrate that SunActive Fe^TM^ entered the bloodstream more rapidly and greatly than FePP, showing peak concentration at 0.5 h for the former versus 2 h for the latter (**Figure 8A**). When biokinetic parameters were calculated (**Table 2**), all kinetic parameters between SunActive Fe^TM^ and FePP were significantly different, showing high C_max_, AUC, T_1/2_, and MRT values, but low T_max_ values for SunActive Fe^TM^. On the other hand, almost similar plasma concentration-time profiles between SunActive Zn^TM^ and ZnO-NPs were found (**Figure 8B**). Biokinetic parameters reveal only slightly high T_1/2_ values for SunActive Zn^TM^ compared to ZnO-NPs, but there were no statistical significances in C_max_, T_max_, AUC, and MRT values between two materials (*p* > 0.05). When oral absorption efficiency was calculated based on AUC values (**Table 2**), absorption of SunActive Fe^TM^ (21.1 ± 4.6%) significantly increased, in comparison with that of FePP (9.7 ± 2.4%), while SunActive Zn^TM^ and ZnO-NPs were determined to have 9.1 ± 0.4 and 8.9 ± 1.0% of oral absorptions, respectively, without statistical difference (*p* > 0.05).

**Table 2:** Biokinetic parameters and oral absorption of SunActive^TM^ products and their counterpart materials after oral administration of a single-dose to rats.

Biokinetic parameters	SunActive Fe^TM^	FePP	SunActive Zn^TM^	ZnO-NP
C_max_ (mg/l)	133.07 ± 6.79^a^	63.53 ± 21.03^b^	13.81 ± 1.67^a^	12.82 ± 1.41^a^
T_max_ (h)	0.5^a^	2^b^	1^a^	1^a^
AUC (h × mg/l)	286.60 ± 62.32^a^	156.97 ± 38.38^b^	153.79 ± 7.32^a^	150.69 ± 16.63^a^
T_1/2_	2.69 ± 1.34^a^	1.01 ± 0.31^b^	7.24 ± 0.14^a^	5.57 ± 0.12^b^
MRT (h)	4.31 ± 1.34^a^	2.49 ± 0.26^b^	11.74 ± 0.36^a^	11.78 ± 0.26^a^
Absorption (%)	21.07 ± 4.58^a^	9.69 ± 2.37^b^	9.05 ± 0.43^a^	8.86 ± 0.98^a^


*Mean values with different superscripts (a,b) in the same row are significantly different between SunActive^*TM*^ product and its counterpart material (*p* < 0.05). Absorption (%) was calculated based on AUC values.*

*FePP, ferric pyrophosphate; C_*max*_, maximum concentration; T_*max*_, time to maximum concentration; AUC, area under the plasma concentration-time curve; T_*1/2*_, half-life; MRT, mean residence time.*

Tissue distributions of SunActive^TM^ products and their counterpart materials were also investigated after a single-dose oral administration to rats. **Figures 8C,D** show no significantly increased Fe or Zn levels in all organs analyzed compared to untreated control groups (*p* > 0.05).

## Discussion

The aim of this study was to investigate the efficacy and potential toxicity of commercially available, surface modified Fe or Zn fortification, coated with low-molecular-carbohydrates (dextrin for SunActive Fe^TM^ and maltodextrin for SunActive Zn^TM^) and glycerides, which enables to have stable and high dispersibility in water, thereby expecting high bioavailability. Hydrodynamic radii obtained from DLS measurement in various media (DDW, MEM, and simulated gastric and intestinal solutions) clearly showed that SunActive^TM^ coating enhanced dipersibility of pristine materials, FePP and ZnO-NPs (**Figures 1B**, **2B**, **3A–C**). However, SunActive^TM^ products measured from SEM showed micron sizes (**Figures 1C**, **2C**), which seems to be attributed to the agglomeration among coating moieties, such as dextrin (for SunActive Fe^TM^) and maltodextrin (SunActive Zn^TM^), during drying process for SEM measurement. The sizes of SunActive Fe^TM^ and SunActive Zn^TM^ can be, therefore, considered to be 240 and 350 nm, respectively, measured by DLS, rather than particle sizes from SEM images. Furthermore, SunActive^TM^ coating ensured long-term dispersion stability, while remarkable sedimentation of non-coated FePP and ZnO-NPs was observed (Supplementary Figure 1). It should be noted that the counterparts, FePP and ZnO-NPs were chosen to have the same primary particle size compared to SunActive^TM^ products. Thus, the reduced hydrodynamic radii of SunActive^TM^ products compared to counterpart materials are attributed to the organic coating, which reduces particle aggregation. It was also found that the hydrodynamic radius and dispersibility were highly dependent on dispersing medium. For instance, SunActive Fe^TM^ and FePP showed significant agglomeration in simulated gastric (**Figure 3B**) and intestinal fluids (**Figure 3C**) compared to DDW (**Figure 1A**) and MEM (**Figure 3A**). The degree of agglomeration was relatively higher for FePP than for SunActive Fe^TM^. Agglomeration was also strongly related to dispersion stability; FePP showed clear sedimentation in DDW and simulated intestinal solution after 48 h of dispersion, while SunActive Fe^TM^ showed retarded sedimentation under all conditions (Supplementary Figure 1). On the other hand, DLS results revealed that SunActive Zn^TM^ showed less agglomeration than ZnO-NPs in MEM (**Figure 3A**) and simulated gastric and intestinal fluids (**Figures 3B,C**). This was in good agreement with sedimentation result (Supplementary Figure 1), which was attributed to the agglomeration of particles in each medium.

The cytotoxicity of SunActive Fe^TM^ was determined to be not affected by surface coating in terms of short- and long-term inhibition of cell proliferation (**Figures 4A,C**) as well as membrane damage (**Figure 5A**). Interestingly, SunActive Fe^TM^ did not inhibit colony-forming ability even after exposure for 7 days, implying its low cytotoxicity. It is worth noting that all biological experiments were performed based on the same Fe or Zn content, in order to compare *in vivo* oral Fe or Zn absorption efficiency of SunActive^TM^ products with that of each counterpart material. Thus, the highest concentrations tested as total compounds for cytotoxicity experiments were 1000 and 320 μg/ml for SunActive Fe^TM^ (8% Fe) and FePP (25% Fe), respectively. Slightly increased intracellular ROS generation was detected in cells exposed to SunActive Fe^TM^ compared to FePP (**Figure 5C**). The slight difference in ROS generation may be related to different particle size in aqueous suspension. Indeed, hydrodynamic radius of SunActive Fe^TM^ was smaller than FePP in MEM (**Figure 3A-a,b**). It is still controversial, but generally accepted that smaller sized NPs induce higher ROS generation (Manke et al., 2013). Nevertheless, overall toxicity results on SunActive Fe^TM^ suggest its low cytotoxicity potential, indicating that low cytotoxic nature of FePP was not influenced by surface coating. On the other hand, SunActive Zn^TM^ exhibited higher inhibition of cell proliferation and colony formation (**Figures 4B,D**), induction of membrane damage (**Figure 5B**), and ROS generation (**Figure 5D**) than ZnO-NPs, when cytotoxicity was expressed based on Zn contents. The IC_50_ values obtained by WST-1 assay were 18.11 and 61.99 μg/ml for SunActive Zn^TM^ and ZnO-NPs as Zn contents, corresponding to 72.45 (25% Zn) and 77.48 μg/ml (80% Zn) as total compounds for the former and the latter, respectively. Hence, the cytotoxicity of SunActive Zn^TM^ is much higher than ZnO-NPs based on Zn content, but similar toxicity between two materials was found when the toxicity was expressed as total compounds. Meanwhile, another critical factor affecting the cytotoxicity is the fate of materials under cell culture condition. It has been well-reported that Zn^2+^ ions cause high toxicity (Song et al., 2010; Yin et al., 2012; Wang et al., 2014; Jo et al., 2016). Furthermore, Zn^2+^ ion release from ZnO-NPs was reported to facilitate ROS generation and LDH release (Song et al., 2010). Indeed, the IC_50_ values for ZnCl_2_ as Zn^2+^ ions were 9.54 μg/ml based on Zn content and 19.89 μg/ml as a total compound (data not shown), which are different from those for SunActive Zn^TM^ or ZnO-NPs. In the present study, SunActive Zn^TM^ was more toxic than ZnO-NPs based on Zn content, in spite of similar Zn dissolution property between two materials in MEM (**Table 1**). Moreover, the solubility of both materials was ∼2% in MEM, indicating their primarily particulate fate. Hence, it is strongly likely that high toxicity of SunActive Zn^TM^ can be related to its small hydrodynamic radius and high dispersibility (**Figure 3A**), but not to their solubility or fate.

When intracellular uptake efficiency was evaluated by measuring total Fe or Zn concentration using ICP-AES, uptake efficiency in both INT-407 and Caco-2 cells was significantly enhanced by SunActive^TM^ coatings, in comparison with their counterpart materials (**Figure 6**). Enhanced cellular uptake of SunActive^TM^ products might be attributed to their reduced hydrodynamic radii compared to counterpart materials (**Figure 3A**), which probably causes high toxicity as well (**Figures 4**, **5**). However, this type of experiment reflects only the amount taken up by cells, which can be different from absorption efficacy through the intestinal epithelium after oral administration. Thus, intestinal transport efficacy and mechanism were also assessed using *in vitro* 3D culture systems, human FAE model and Caco-2 monolayers. The FAE model represents M cells found in the Peyer’s patches, which involve in the intestinal transport of a variety of materials and immune system (Kernéis et al., 1997; Neutra et al., 2001; Jang et al., 2004). Caco-2 monolayers were often used to represent a dense network of intestinal tight junctions (Ma et al., 2005; Hubatsch et al., 2007). The results show that both SunActive Fe^TM^ and SunActive Zn^TM^ were primarily transported by M cells, but slight transports by Caco-2 monolayers were also found (**Figure 7**). FePP and ZnO-NPs were also found to be translocated in the same manner. In other words, intestinal transport mechanism was not affected by surface coating. Since *in vivo* intestinal transport occurs simultaneously through M cells and tight junctions, it is more reasonable that combined translocation amounts obtained by two different models are compared. As shown in **Figures 7C,D**, significantly high intestinal transport of SunActive Fe^TM^ compared to FePP suggests that surface coating can contribute to enhance intestinal transport of mineral fortification. However, increased translocation of SunActive Zn^TM^ compared to ZnO-NPs was not found. These results indicate that surface modification does not always result in transport efficiency, and that the intestinal transport of materials is governed by various factors, such as particle size, dispersibility, and coating stability.

Plasma concentration-time profiles after a single-dose oral administration to rats were highly consistent with the intestinal transport results. SunActive Fe^TM^ remarkably increased oral absorption rate and absorption efficiency, as shown in rapid T_max_ and high AUC values (**Figure 8A** and **Table 2**). Oral absorption efficacy of SunActive Fe^TM^ significantly increased by about 2.2 fold compared to its counterpart material, FePP. This result indicates that surface coating by dextrin and glycerides can enhance oral Fe bioavailability. This seems to be strongly associated with hydrodynamic radius, dispersibility, and solubility. SunActive Fe^TM^ had smaller hydrodynamic radius than FePP in all media tested (**Figure 3C-a,b**), showing better dispersibility than FePP (Supplementary Figure 1), which is likely to facilitate oral absorption of Fe moiety. Furthermore, intestinal solubility of SunActive Fe^TM^ dramatically enhanced compared to FePP, and thus, total gastrointestinal solubility of the former significantly increased in comparison with the latter, which surely affects Fe bioavailability (Zariwala et al., 2013). Meanwhile, almost similar biokinetic behaviors between SunActive Zn^TM^ and ZnO-NPs were found, as observed by similar plasma concentration versus time curves and biokinetic parameters (**Figure 8B** and **Table 2**). Moreover, oral absorption of SunActive Zn^TM^ did not significantly increase (**Table 2**), although reduced hydrodynamic radius and enhanced dispersibility compared to ZnO-NPs were observed (**Figure 3** and Supplementary Figure 1). It should be noted that major surface coating materials for SunActive Fe^TM^ and SunActive Zn^TM^ are dextrin (54.7%) and maltodextrin (62.1%), respectively, and maltodextrin is more easily digestible than dextrin and rapidly absorbed as glucose (Chronakis, 1998). It is, therefore, probable that SunActive Zn^TM^ is easily degradable after oral administration, resulting in its similar oral absorption compared to ZnO-NPs. Contrary to SunActive Zn^TM^, SunActive Fe^TM^ with dextrin coating tends to be less degraded under gastrointestinal condition, which can contribute to enhance Fe bioavailability. It is interesting to note that SunActive Zn^TM^ had similar hydrodynamic radius compared to ZnO-NPs in simulated gastric fluid, while significantly reduced hydrodynamic radius was observed for SunActive Fe^TM^ compared to FePP (**Figure 3B**). Moreover, no significant difference in total gastrointestinal solubility between SunActive Zn^TM^ and ZnO-NPs was found (**Table 1**), which may explain similar oral Zn absorption between two materials. On the other hand, much higher total gastrointestinal solubility of SunActive Zn^TM^ than SunActive Fe^TM^ (**Table 1**) might also support coating stability of the latter. It is worth noting that biokinetic study was performed to have an equivalent amount of Fe or Zn, based on Fe or Zn contents in all tested materials. Taken together, it is likely that oral absorption of Fe or Zn fortification can be enhanced by surface coating to some extent, but highly dependent on the nature of fortified minerals and surface coating materials. High oral absorption efficacy of SunActive Fe^TM^ is likely to be attributed to its increased intestinal transport through M cells and Caco-2 monolayers, which might be related to the size, dispersibility, and coating stability. Interestingly, we found that surface coating did not affect tissue distribution patterns, showing no accumulation in all organs analyzed after a single-dose oral administration of both SunActive^TM^ products. It can be strongly suggested that enhanced oral Fe absorption by surface coating did not influence on tissue accumulation, and surface modified Fe or Zn fortification has low toxicity potential.

## Conclusion

Surface modified Fe or Zn fortification with low-molecular-carbohydrates and glycerides had stable and high dispersibility as well as small hydrodynamic radius in various aqueous media. However, cytotoxicity, uptake behaviors, and oral absorption were highly dependent on the nature of fortified minerals and surface coating materials; SunActive Fe^TM^ with dextrin coating enhanced cellular uptake, intestinal transport efficacy, and bioavailability, but did not cause cytotoxicity, which was not found for SunActive Zn^TM^ with maltodextrin coating. The discrepancy can be attributed to the stability of coating materials under physiological condition. On the other hand, surface modification of Fe or Zn fortification did not affect intestinal transport mechanism and tissue distribution pattern. Taken together, bioavailability of mineral fortification can be enhanced by surface modification to some extent, without affecting potential toxicity. These findings will provide a useful strategy to enhance oral absorption efficacy of food fortification at safe levels.

## Author Contributions

Conceived and designed the experiments and wrote the manuscript: S-JC and J-MO, performed biological experiments: H-JiK, S-HB, performed physicochemical experiments: H-JuK, K-MK, JHS, performed statistical and data analysis: M-RG and JY.

## Conflict of Interest Statement

The authors declare that the research was conducted in the absence of any commercial or financial relationships that could be construed as a potential conflict of interest.
