# Supplementary material for: Cytotoxicity, Intestinal Transport, and Bioavailability of Dispersible Iron and Zinc Supplements
Source: Front Microbiol. 2017 Apr 28;8:749. doi: 10.3389/fmicb.2017.00749 (PMC5408065; doi:10.3389/fmicb.2017.00749)
Supplement: Supplementary file 1 [file Presentation_1.PDF]

frontiers

Supplementary Material

Cytotoxicity, intestinal transport, and bioavailability of dispersible iron and zinc supplements

Hyeon-Jin Kim, Song-Hwa Bae, Hyoung-Jun Kim, Kyoung-Min Kim, Jaeho Song, Mi-Ran Go, Jin Yu, Jae-Min Oh*, and Soo-Jin Choi*

*** Correspondence:** Jae-Min Oh: jaemin.oh@yonsei.ac.kr

Soo-Jin Choi: sjchoi@swu.ac.kr

1 Supplementary Figure

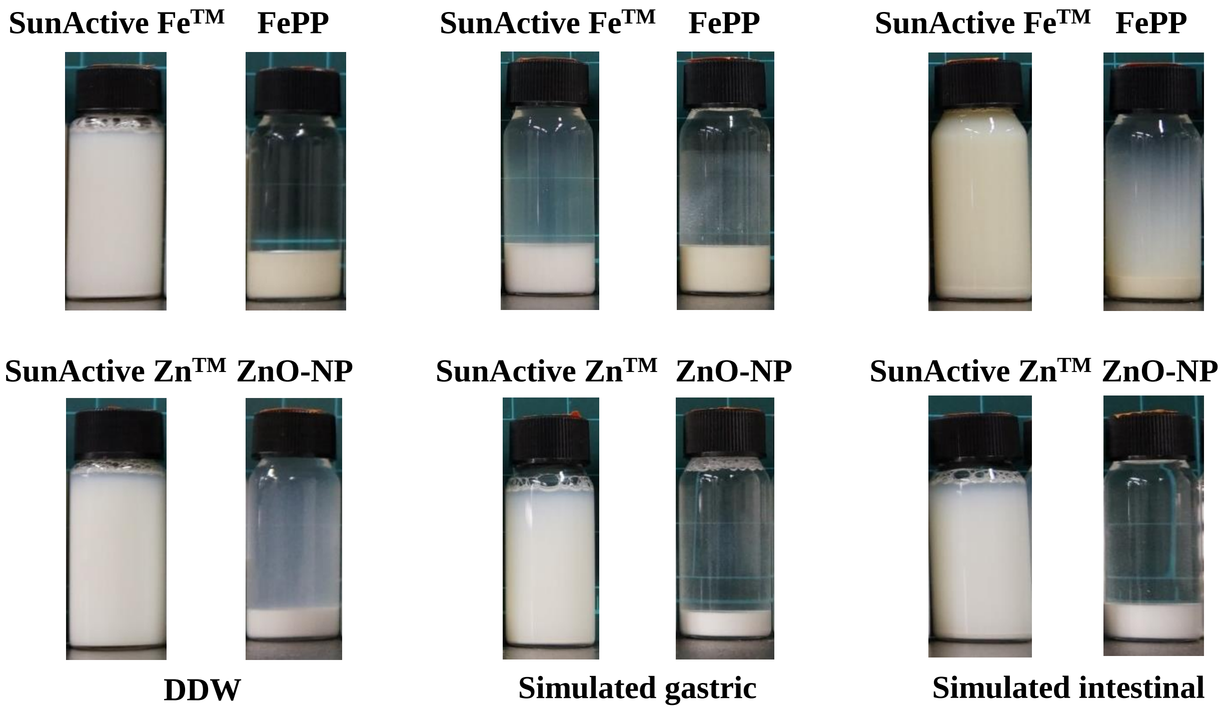

Supplementary Figure 1. Dispersion stability of each sample (5%) in various media after 48 h of dispersion.
